# Early neurological outcome prediction model after bystander-witnessed out-of-hospital cardiac arrest: a nationwide population-based study

**DOI:** 10.1186/cc10885

**Published:** 2012-03-20

**Authors:** Y Goto, T Maeda, Y Goto, H Inaba

**Affiliations:** 1 Kanazawa University Hospital, Kanazawa, Japan; 2 Yawata Medical Center, Komatsu, Japan; 3 Kanazawa University Graduate School of Medicine, Kanazawa, Japan

## Introduction

Identification of prehospital prognostic factors in out-of-hospital cardiac arrests (OHCAs) with prehospital return of spontaneous circulation (ROSC) and establishment of a prediction model for survival with favorable neurological outcome may minimize the costs and save the medical resources. In this study, we developed a best model for predicting 1-month survival with favorable neurological outcome (defined as Glasgow-Pittsburgh cerebral performance category (CPC) scale = 1 or 2), using a logistic regression analysis.

## Methods

Of 522,801 resuscitation-attempted adult patients after OHCAs, 9,876 bystander-witnessed arrests of presumed cardiac origin with prehospital ROSC were analyzed in a prospectively recorded nationwide Utstein-style database in Japan over 5 years (2005 to 2009). The endpoint was 1-month survival with favorable neurological outcome. We performed multivariate logistic regression analysis to develop a prediction model using the prehospital factors.

## Results

Overall rates of 1-month survival and that with favorable neurological outcome were 56.7% (*n *= 5,604) and 40.6% (*n *= 4,013), respectively. Multivariate logistic regression analysis revealed that the odds ratio for age, shockable initial rhythm and collapse-ROSC time interval were 0.964 (95% CI 0.961 to 0.967), 3.564 (95% CI 3.232 to 3.934) and 0.967 (95% CI 0.963 to 0.970), respectively, and that these variables were identified as the best variables for developing a prediction model. A statistical outcome prediction model using these three variables was as follows: Pf = exp(B)/[1 + exp(B)], where Pf is the probability of a favorable outcome and exp(B) is the exponential function of B: B = -0.037 × age (years) + 0.635 × (shockable rhythm (1 or 0)) - 0.034 × (collapse-ROSC time interval (minutes)) + 2.540. The area under the receiver operating characteristic curve of this model for predicting 1-month favourable neurological outcome was 0.764.

## Conclusion

Three prehospital prognostic factors (age, shockable initial rhythm and collapse-ROSC time interval) were identified as the best variables in predicting favorable neurological outcomes in OHCAs with prehospital ROSC. A model using these prehospital prognostic factors has shown a good predictive value for estimating 1-month survival with favorable neurological outcome in OHCA patients. Although this novel model needs to be validated using another external dataset, this model may help to minimize the cost and save medical resources.

